# Supplementary material for: The Gut Bacteria Dysbiosis Contributes to Chronic Graft-Versus-Host Disease Associated With a Treg/Th1 Ratio Imbalance
Source: Front Microbiol. 2022 Sep 8;13:813576. doi: 10.3389/fmicb.2022.813576 (PMC9493085; doi:10.3389/fmicb.2022.813576)
Supplement: Supplementary file 1 [file Data_Sheet_1.zip › P101SC18090073-01-B1-3-4_result/05.WebShow/alpha_rarefaction_plots/rarefaction_plots.html]

Rarefaction Curves


|  |  |  |  |
| --- | --- | --- | --- |
| **Select a Metric:** | PD\_whole\_tree chao1 observed\_species shannon | **Select a Category:** | Description SampleID |

  

**Show Categories:
 
All
None
Invert**

**Legend**

|  |  |  |  |
| --- | --- | --- | --- |
| ▶ |  | ■ | **MC** |
| ∟ |  | ◆ | **MC1** |
| ∟ |  | ◆ | **MC2** |
| ∟ |  | ◆ | **MC3** |
| ∟ |  | ◆ | **MC4** |
| ∟ |  | ◆ | **MC5** |
| ▶ |  | ■ | **MD** |
| ∟ |  | ◆ | **MD1** |
| ∟ |  | ◆ | **MD2** |
| ∟ |  | ◆ | **MD3** |
| ∟ |  | ◆ | **MD4** |
| ∟ |  | ◆ | **MD5** |
| ▶ |  | ■ | **MC1** |
| ∟ |  | ◆ | **MC1** |
| ▶ |  | ■ | **MC2** |
| ∟ |  | ◆ | **MC2** |
| ▶ |  | ■ | **MC3** |
| ∟ |  | ◆ | **MC3** |
| ▶ |  | ■ | **MC4** |
| ∟ |  | ◆ | **MC4** |
| ▶ |  | ■ | **MC5** |
| ∟ |  | ◆ | **MC5** |
| ▶ |  | ■ | **MD1** |
| ∟ |  | ◆ | **MD1** |
| ▶ |  | ■ | **MD2** |
| ∟ |  | ◆ | **MD2** |
| ▶ |  | ■ | **MD3** |
| ∟ |  | ◆ | **MD3** |
| ▶ |  | ■ | **MD4** |
| ∟ |  | ◆ | **MD4** |
| ▶ |  | ■ | **MD5** |
| ∟ |  | ◆ | **MD5** |
| ▶ |  | ■ | **MC** |
| ∟ |  | ◆ | **MC1** |
| ∟ |  | ◆ | **MC2** |
| ∟ |  | ◆ | **MC3** |
| ∟ |  | ◆ | **MC4** |
| ∟ |  | ◆ | **MC5** |
| ▶ |  | ■ | **MD** |
| ∟ |  | ◆ | **MD1** |
| ∟ |  | ◆ | **MD2** |
| ∟ |  | ◆ | **MD3** |
| ∟ |  | ◆ | **MD4** |
| ∟ |  | ◆ | **MD5** |
| ▶ |  | ■ | **MC1** |
| ∟ |  | ◆ | **MC1** |
| ▶ |  | ■ | **MC2** |
| ∟ |  | ◆ | **MC2** |
| ▶ |  | ■ | **MC3** |
| ∟ |  | ◆ | **MC3** |
| ▶ |  | ■ | **MC4** |
| ∟ |  | ◆ | **MC4** |
| ▶ |  | ■ | **MC5** |
| ∟ |  | ◆ | **MC5** |
| ▶ |  | ■ | **MD1** |
| ∟ |  | ◆ | **MD1** |
| ▶ |  | ■ | **MD2** |
| ∟ |  | ◆ | **MD2** |
| ▶ |  | ■ | **MD3** |
| ∟ |  | ◆ | **MD3** |
| ▶ |  | ■ | **MD4** |
| ∟ |  | ◆ | **MD4** |
| ▶ |  | ■ | **MD5** |
| ∟ |  | ◆ | **MD5** |
| ▶ |  | ■ | **MC** |
| ∟ |  | ◆ | **MC1** |
| ∟ |  | ◆ | **MC2** |
| ∟ |  | ◆ | **MC3** |
| ∟ |  | ◆ | **MC4** |
| ∟ |  | ◆ | **MC5** |
| ▶ |  | ■ | **MD** |
| ∟ |  | ◆ | **MD1** |
| ∟ |  | ◆ | **MD2** |
| ∟ |  | ◆ | **MD3** |
| ∟ |  | ◆ | **MD4** |
| ∟ |  | ◆ | **MD5** |
| ▶ |  | ■ | **MC1** |
| ∟ |  | ◆ | **MC1** |
| ▶ |  | ■ | **MC2** |
| ∟ |  | ◆ | **MC2** |
| ▶ |  | ■ | **MC3** |
| ∟ |  | ◆ | **MC3** |
| ▶ |  | ■ | **MC4** |
| ∟ |  | ◆ | **MC4** |
| ▶ |  | ■ | **MC5** |
| ∟ |  | ◆ | **MC5** |
| ▶ |  | ■ | **MD1** |
| ∟ |  | ◆ | **MD1** |
| ▶ |  | ■ | **MD2** |
| ∟ |  | ◆ | **MD2** |
| ▶ |  | ■ | **MD3** |
| ∟ |  | ◆ | **MD3** |
| ▶ |  | ■ | **MD4** |
| ∟ |  | ◆ | **MD4** |
| ▶ |  | ■ | **MD5** |
| ∟ |  | ◆ | **MD5** |
| ▶ |  | ■ | **MC** |
| ∟ |  | ◆ | **MC1** |
| ∟ |  | ◆ | **MC2** |
| ∟ |  | ◆ | **MC3** |
| ∟ |  | ◆ | **MC4** |
| ∟ |  | ◆ | **MC5** |
| ▶ |  | ■ | **MD** |
| ∟ |  | ◆ | **MD1** |
| ∟ |  | ◆ | **MD2** |
| ∟ |  | ◆ | **MD3** |
| ∟ |  | ◆ | **MD4** |
| ∟ |  | ◆ | **MD5** |
| ▶ |  | ■ | **MC1** |
| ∟ |  | ◆ | **MC1** |
| ▶ |  | ■ | **MC2** |
| ∟ |  | ◆ | **MC2** |
| ▶ |  | ■ | **MC3** |
| ∟ |  | ◆ | **MC3** |
| ▶ |  | ■ | **MC4** |
| ∟ |  | ◆ | **MC4** |
| ▶ |  | ■ | **MC5** |
| ∟ |  | ◆ | **MC5** |
| ▶ |  | ■ | **MD1** |
| ∟ |  | ◆ | **MD1** |
| ▶ |  | ■ | **MD2** |
| ∟ |  | ◆ | **MD2** |
| ▶ |  | ■ | **MD3** |
| ∟ |  | ◆ | **MD3** |
| ▶ |  | ■ | **MD4** |
| ∟ |  | ◆ | **MD4** |
| ▶ |  | ■ | **MD5** |
| ∟ |  | ◆ | **MD5** |

|  |  |  |  |  |  |  |  |  |  |
| --- | --- | --- | --- | --- | --- | --- | --- | --- | --- |
| Description | Seqs/Sample | PD\_whole\_tree Ave. | PD\_whole\_tree Err. | chao1 Ave. | chao1 Err. | observed\_species Ave. | observed\_species Err. | shannon Ave. | shannon Err. |
| MC | 10.0 | 2.103 | 0.075 | 28.290 | 2.706 | 8.700 | 0.141 | 3.036 | 0.036 || MC | 11798.0 | 20.826 | 1.036 | 429.148 | 18.663 | 354.660 | 19.092 | 6.156 | 0.283 || MC | 23586.0 | 23.307 | 1.063 | 464.133 | 13.979 | 409.620 | 14.991 | 6.168 | 0.283 || MC | 35374.0 | 24.523 | 1.247 | 476.389 | 18.755 | 435.920 | 17.508 | 6.173 | 0.287 || MC | 47162.0 | 25.259 | 1.426 | 481.930 | 20.044 | 451.900 | 16.837 | 6.177 | 0.286 || MC | 58950.0 | 25.778 | 1.494 | 484.468 | 17.644 | 462.660 | 17.107 | 6.178 | 0.285 || MC | 70738.0 | 26.096 | 1.519 | 485.974 | 20.016 | 469.600 | 17.625 | 6.180 | 0.284 || MD | 10.0 | 1.988 | 0.108 | 32.080 | 2.664 | 9.140 | 0.102 | 3.147 | 0.020 || MD | 11798.0 | 21.192 | 1.023 | 456.563 | 18.131 | 379.660 | 25.415 | 6.498 | 0.331 || MD | 23586.0 | 23.647 | 1.101 | 485.255 | 20.999 | 433.400 | 23.129 | 6.515 | 0.332 || MD | 35374.0 | 24.917 | 1.079 | 500.411 | 17.919 | 461.080 | 20.849 | 6.520 | 0.331 || MD | 47162.0 | 25.576 | 1.053 | 509.346 | 18.479 | 477.860 | 20.326 | 6.524 | 0.333 || MD | 58950.0 | 26.053 | 1.017 | 509.975 | 15.601 | 488.140 | 19.385 | 6.526 | 0.332 || MD | 70738.0 | 26.394 | 0.972 | 514.382 | 14.788 | 495.400 | 18.489 | 6.527 | 0.333 |
| SampleID | Seqs/Sample | PD\_whole\_tree Ave. | PD\_whole\_tree Err. | chao1 Ave. | chao1 Err. | observed\_species Ave. | observed\_species Err. | shannon Ave. | shannon Err. |
| MC1 | 10.0 | 2.176 | nan | 32.600 | nan | 8.700 | nan | 3.019 | nan || MC1 | 11798.0 | 21.024 | nan | 424.131 | nan | 352.000 | nan | 5.998 | nan || MC1 | 23586.0 | 23.383 | nan | 465.332 | nan | 405.400 | nan | 6.007 | nan || MC1 | 35374.0 | 24.213 | nan | 470.517 | nan | 428.100 | nan | 6.002 | nan || MC1 | 47162.0 | 25.030 | nan | 472.908 | nan | 442.500 | nan | 6.012 | nan || MC1 | 58950.0 | 25.484 | nan | 476.432 | nan | 454.900 | nan | 6.012 | nan || MC1 | 70738.0 | 25.767 | nan | 476.512 | nan | 462.000 | nan | 6.014 | nan || MC2 | 10.0 | 2.135 | nan | 27.300 | nan | 8.800 | nan | 3.062 | nan || MC2 | 11798.0 | 19.171 | nan | 404.042 | nan | 322.200 | nan | 5.905 | nan || MC2 | 23586.0 | 22.174 | nan | 439.727 | nan | 385.100 | nan | 5.916 | nan || MC2 | 35374.0 | 23.569 | nan | 449.483 | nan | 409.600 | nan | 5.920 | nan || MC2 | 47162.0 | 24.135 | nan | 454.857 | nan | 428.400 | nan | 5.925 | nan || MC2 | 58950.0 | 24.887 | nan | 461.454 | nan | 439.200 | nan | 5.923 | nan || MC2 | 70738.0 | 25.261 | nan | 456.234 | nan | 445.000 | nan | 5.926 | nan || MC3 | 10.0 | 1.960 | nan | 24.200 | nan | 8.500 | nan | 3.007 | nan || MC3 | 11798.0 | 22.263 | nan | 449.296 | nan | 359.900 | nan | 5.924 | nan || MC3 | 23586.0 | 25.111 | nan | 477.202 | nan | 419.600 | nan | 5.938 | nan || MC3 | 35374.0 | 26.881 | nan | 506.612 | nan | 456.200 | nan | 5.948 | nan || MC3 | 47162.0 | 27.973 | nan | 515.794 | nan | 474.700 | nan | 5.944 | nan || MC3 | 58950.0 | 28.657 | nan | 514.893 | nan | 488.200 | nan | 5.952 | nan || MC3 | 70738.0 | 29.058 | nan | 515.812 | nan | 497.000 | nan | 5.952 | nan || MC4 | 10.0 | 2.108 | nan | 28.550 | nan | 8.900 | nan | 3.094 | nan || MC4 | 11798.0 | 21.346 | nan | 451.888 | nan | 381.600 | nan | 6.640 | nan || MC4 | 23586.0 | 23.576 | nan | 478.081 | nan | 429.700 | nan | 6.651 | nan || MC4 | 35374.0 | 24.527 | nan | 484.134 | nan | 454.500 | nan | 6.664 | nan || MC4 | 47162.0 | 25.104 | nan | 487.796 | nan | 467.100 | nan | 6.664 | nan || MC4 | 58950.0 | 25.460 | nan | 489.050 | nan | 475.100 | nan | 6.663 | nan || MC4 | 70738.0 | 25.612 | nan | 497.550 | nan | 480.000 | nan | 6.664 | nan || MC5 | 10.0 | 2.138 | nan | 28.800 | nan | 8.600 | nan | 2.998 | nan || MC5 | 11798.0 | 20.325 | nan | 416.381 | nan | 357.600 | nan | 6.313 | nan || MC5 | 23586.0 | 22.289 | nan | 460.322 | nan | 408.300 | nan | 6.327 | nan || MC5 | 35374.0 | 23.423 | nan | 471.202 | nan | 431.200 | nan | 6.334 | nan || MC5 | 47162.0 | 24.052 | nan | 478.291 | nan | 446.800 | nan | 6.339 | nan || MC5 | 58950.0 | 24.401 | nan | 480.509 | nan | 455.900 | nan | 6.340 | nan || MC5 | 70738.0 | 24.784 | nan | 483.760 | nan | 464.000 | nan | 6.340 | nan || MD1 | 10.0 | 1.888 | nan | 36.400 | nan | 9.300 | nan | 3.174 | nan || MD1 | 11798.0 | 20.948 | nan | 471.622 | nan | 399.100 | nan | 6.761 | nan || MD1 | 23586.0 | 23.832 | nan | 508.580 | nan | 453.100 | nan | 6.776 | nan || MD1 | 35374.0 | 25.054 | nan | 512.368 | nan | 477.100 | nan | 6.783 | nan || MD1 | 47162.0 | 25.772 | nan | 526.859 | nan | 495.700 | nan | 6.788 | nan || MD1 | 58950.0 | 26.253 | nan | 523.857 | nan | 505.600 | nan | 6.788 | nan || MD1 | 70738.0 | 26.645 | nan | 529.029 | nan | 513.000 | nan | 6.791 | nan || MD2 | 10.0 | 2.087 | nan | 30.600 | nan | 9.100 | nan | 3.142 | nan || MD2 | 11798.0 | 21.929 | nan | 468.532 | nan | 397.400 | nan | 6.701 | nan || MD2 | 23586.0 | 24.212 | nan | 493.269 | nan | 447.600 | nan | 6.728 | nan || MD2 | 35374.0 | 25.577 | nan | 512.296 | nan | 474.000 | nan | 6.726 | nan || MD2 | 47162.0 | 26.221 | nan | 521.403 | nan | 490.800 | nan | 6.734 | nan || MD2 | 58950.0 | 26.694 | nan | 520.929 | nan | 499.500 | nan | 6.737 | nan || MD2 | 70738.0 | 27.009 | nan | 525.833 | nan | 506.000 | nan | 6.737 | nan || MD3 | 10.0 | 2.127 | nan | 30.600 | nan | 9.100 | nan | 3.142 | nan || MD3 | 11798.0 | 22.589 | nan | 464.663 | nan | 394.500 | nan | 6.678 | nan || MD3 | 23586.0 | 25.109 | nan | 492.090 | nan | 448.000 | nan | 6.692 | nan || MD3 | 35374.0 | 26.331 | nan | 510.820 | nan | 475.400 | nan | 6.703 | nan || MD3 | 47162.0 | 26.930 | nan | 513.292 | nan | 489.200 | nan | 6.702 | nan || MD3 | 58950.0 | 27.311 | nan | 515.273 | nan | 499.500 | nan | 6.704 | nan || MD3 | 70738.0 | 27.493 | nan | 515.216 | nan | 505.000 | nan | 6.705 | nan || MD4 | 10.0 | 1.991 | nan | 29.000 | nan | 9.000 | nan | 3.114 | nan || MD4 | 11798.0 | 19.577 | nan | 421.809 | nan | 331.700 | nan | 5.862 | nan || MD4 | 23586.0 | 21.791 | nan | 445.905 | nan | 390.500 | nan | 5.877 | nan || MD4 | 35374.0 | 23.133 | nan | 465.591 | nan | 422.000 | nan | 5.885 | nan || MD4 | 47162.0 | 23.829 | nan | 474.175 | nan | 440.000 | nan | 5.884 | nan || MD4 | 58950.0 | 24.319 | nan | 480.380 | nan | 452.100 | nan | 5.890 | nan || MD4 | 70738.0 | 24.653 | nan | 487.053 | nan | 461.000 | nan | 5.889 | nan || MD5 | 10.0 | 1.848 | nan | 33.800 | nan | 9.200 | nan | 3.162 | nan || MD5 | 11798.0 | 20.918 | nan | 456.187 | nan | 375.600 | nan | 6.490 | nan || MD5 | 23586.0 | 23.289 | nan | 486.432 | nan | 427.800 | nan | 6.501 | nan || MD5 | 35374.0 | 24.487 | nan | 500.980 | nan | 456.900 | nan | 6.504 | nan || MD5 | 47162.0 | 25.125 | nan | 511.003 | nan | 473.600 | nan | 6.509 | nan || MD5 | 58950.0 | 25.687 | nan | 509.437 | nan | 484.000 | nan | 6.510 | nan || MD5 | 70738.0 | 26.172 | nan | 514.778 | nan | 492.000 | nan | 6.510 | nan |
